# PPAR δ inhibition protects against palmitic acid-LPS induced lipidosis and injury in cultured hepatocyte L02 cell

**DOI:** 10.7150/ijms.37677

**Published:** 2019-10-21

**Authors:** Yi Li, Chenwei Wang, Jiyuan Lu, Ke Huang, Yu Han, Junlin Chen, Yan Yang, Bin Liu

**Affiliations:** 1 School/Hospital of Stomatology, Lanzhou University, Lanzhou, China; 2 College of Life Science & Technology, Huazhong University of Science and Technology, Wuhan, China; 3 Department of Endocrinology, Gansu Provincial Hospital, Lanzhou, China

**Keywords:** Non-alcoholic fatty liver disease, PPAR δ, L02 Cell

## Abstract

**Background:** Non-alcoholic fatty liver disease (NAFLD) is the most common chronic liver disease, and its pathogenesis and mechanism are intricate. In the present study, we aimed to evaluate the role of PPAR δ in LPS associated NAFLD and to investigate the signal transduction pathways underlying PPAR δ treatment in vitro. **Material and Methods:** L02 cells were exposed to palmitic acid (PA) and/or LPS in the absence or presence of PPAR δ inhibition and/or activation. **Results:** LPS treatment markedly increased lipid deposition, FFA contents, IL-6 and TNF-α levels, and cell apoptosis in PA treatment (NAFLD model). PPAR δ inhibition protects L02 cells against LPS-induced lipidosis and injury. Conversely, the result of PPAR δ activation showed the reverse trend. LPS+PA treatment group significantly decreases the relative expression level of IRS-1, PI3K, AKT, phosphorylation of AKT, TLR-4, MyD88, phosphorylation of IKKα, NF-κB, Bcl-2 and increases the relative expression level of Bax, cleaved caspase 3 and cleaved caspase 8, compared with the cells treated with NAFLD model. PPAR δ inhibition upregulated the related proteins' expression level in insulin resistance and inflammation pathway and downregulated apoptotic relevant proteins. Instead, PPAR δ agonist showed the reverse trend. **Conclusion:** Our data show that PPAR δ inhibition reduces steatosis, inflammation and apoptosis in LPS-related NAFLD damage, in vitro. PPAR δ may be a potential therapeutic implication for NAFLD.

## Introduction

Non-alcoholic fatty liver disease (NAFLD) is the most common chronic liver disease in many countries 1. NAFLD is characterized by a wide spectrum of manifestations ranging from simple steatosis, non-alcoholic steatohepatitis (NASH) to advanced fibrosis and may evolve to liver cirrhosis and hepatocellular carcinoma (HCC) 2-5. NAFLD is becoming the most common liver disease affecting liver transplantation 6. Although many different pharmacotherapeutic strategies have been used in clinic, such as thiazolidinediones, vitamin E, losartan and silybin taking orally, the impact of these therapies is still not satisfactory 7-10. Therefore, further study on the pathogenesis and mechanism of NAFLD will be beneficial in the treatment of NAFLD.

It is generally recognized that the causes of NAFLD are associated with heredity, diet, insulin resistance (IR) and adipokine, as well as potential factors which need to be further verified, including endocrine disruptors (such as bisphenol A, phthalates) and ecological disorders of intestinal flora 11-16. Recent studies suggest that the gut microbiota contributes to metabolic liver disease 17-19. It has been well established that gut microbiota controls the gut barrier function and, therefore, the progression of 'metabolic endotoxaemia' characterised by the translocation of specific microbe-associated molecular patterns such as lipopolysaccharides (LPS) into the systemic circulation 20-23. Gut-derived bacterial LPS is brought to the liver by the portal circulation, which combines with Toll-like receptor 4 (TLR-4) complexes on liver cell surface and induces the production of inflammatory cytokines 24-26. LPS might result in low-grade chronic inflammation and thereby cause insulin resistance, contributing to the development of NAFLD 27-30. However, the role of LPS in development of NAFLD has not been fully elucidated.

Peroxisome proliferator-activated receptor δ (PPAR δ) is a metabolic regulator, with biological functions in skeletal muscle, fat, intestine, liver and the heart 31. Evidences suggest that PPAR δ activation enhances free fatty acids (FFAs) transport and oxidation, improves glucose homeostasis through improvement of insulin sensitivity and inhibition of glucose output, attenuates macrophage inflammatory responses, and increases plasma high-density lipoprotein (HDL) concentrations 32-35. In the mouse model of NASH, PPAR δ improves hepatic steatosis and inflammation by regulation of lipid metabolism and inhibition of inflammatory response 36. By contrast, little is known about the role of PPAR δ during NAFLD with a high LPS level.

Therefore, we hypothesized that LPS play a role in development of NAFLD and that PPAR δ may be a key factor during the process. The aim of this study is to explore the effect of LPS in NAFLD and investigate whether PPAR δ could exert influence in the lipid deposition and inflammatory response relating to LPS *in vitro.*

## Materials and Methods

### Cell Culture

Human hepatocyte L02 cell line was obtained from the Chinese Academy of Sciences Cell Bank (Shanghai, China) and cultured in Roswell Park Memorial Institute (RPMI) 1640 Medium (Gibco, Grand Island, NY, USA) supplemented with 10% (v/v) fetal bovine serum (Invitrogen, Carlsbad, CA, USA), 100 U/mL penicillin and 100 μg /mL streptomycin in 5% CO2 at 37°C. The medium was changed every 2-3 days, and the cells were passaged for subculturing or subsequent experiments when they grew to 80% confluence.

### Targeted PPAR δ inhibition and activation

For PPAR δ activation, we use 0.5 μmol/L GW0742 (Selleck Chemicals, Houston, TX, USA), a PPAR δ-selective agonist, for 24h. For PPAR δ inhibition, the siRNA target sequence used for PPAR δ knockdown (si-PPAR δ). The PPAR δ-specific siRNA gene sequence was 5'-GCAAACCCUUCAGUGAUAUTT -3' and the gene sequence of negative control siRNA (N.C. siRNA) was 5'-UUCUCCGAACGUGUCACGUTT -3'. PPAR δ siRNA and N.C. siRNA was purchased from GenePharma (GenePharma, Shanghai, China). L02 cells were used transfected with 20 pmol of N.C. siRNA or PPAR δ-specific siRNA, using Lipofectamine 2000 and Opti-MEM media (Life Technologies, Gaithersburg, MD, USA) after seeding cells attached to the bottom. After 24h PPAR δ activation or transfection, L02 cells were harvested and immediately subjected to isolation of total RNA and protein. For confirming the PPAR δ knockdown and over expression efficiency, real-time-PCR and Western blot analyses were performed on the isolated total RNA and protein, respectively. Negative control siRNA (N.C. siRNA) or PPAR δ siRNA-transfected L02 cells were incubated in 0.4mM palmitic acid or/and 800ng/ml LPS for 24h. L02 cells were exposed to 0.4mM palmitic acid (PA) or (and) 800ng/ml LPS with or without 1nM GW0742 treatment.

### Hoechst 33342 Staining Assay

Cells grown on a sterile cover slip in twelve-well plates were treated with different concentrations of the test compound for 24 h. The culture medium containing compounds was then removed, and the cells were fixed in 4% paraformaldehyde for 10 min at room temperature. After being washed twice with phosphate buffer saline (PBS), the cells were stained with 0.5 mL of 10μg/mL Hoechst 33342 (Invitrogen, Eugene, OR, USA) for 10 min at room temperature. The coverslips were then washed three times with PBS, placed onto glass slides, and covered with mounting medium. The stained nuclei were observed under a fluorescence microscope (Olympus, Tokyo, Japan).

### Oil Red O Staining

Cells cultured on sterile cover slips in twelve-well plates were treated with different concentrations of the test compound for 24 h. The oil red O staining (Nanjing Jiancheng Bioengineering Institute, Nanjing, China) was conducted based on a standard protocol as follows. Cultured cells were washed three times with PBS and fixed with 4% formaldehyde for 10 min at room temperature. Subsequently, cells were stained with oil red O working solution for 15 min at room temperature and washed three times with ddH_2_O. The nuclear was stained with hematoxylin for another 5 min. The cover slips were then washed three times with ddH_2_O, placed onto glass slides, and covered with mounting medium. The images were recorded under a light microscope (OLYMPUS) at 400× magnification.

### FFA content measurement

The FFA content measurement kit was obtained from Nanjing Jiancheng Bioengineering Institute (Nanjing, China). Cells were seeded in a density of 10^6^ cells/culture-flasks either for control group (containing only medium with supplements) or each treated group for 24h. Then collected the supernatant for each group after 5, 000 rpm centrifugation. The free fatty acid assay was performed according to the manufacturer's instructions.

### Annexin V-FITC/ propidium iodide (PI) double staining

According to the manufacturer's instructions of annexin-V FITC apoptosis kit (BD, San Jose, CA, USA), cells were cultivated in a density of 10^6^ cells/culture-flasks either for control group (containing only medium with supplements) or other treated groups for 24h. Then, the cells were collected and washed twice with PBS and then resuspended in 100 µL 1 × binding buffer. The cells were subjected to 5 µL of FITC Annexin V and 5 µL PI staining and incubated for 30 min at room temperature in the dark. Afterward, 100µL 1× binding buffer were added. The apoptosis ratio was quantified by system software (Cell Quest; BD Biosciences, Becton Dickinson FACS AriaIII, NY, USA).

### The relative expression level of IL-6 and TNF-α

The expression level of IL-6 and TNF-α were measured by commercially available enzyme-linked immunosorbent assays kit (Neobioscience technology company, Beijing, China) according to the manufacturer's protocol. All values were expressed as the mean of the three determinations. The concentration was determined by standard curve. All analyses were performed at least three times for each individual cell-stimulation assay.

### Western blot analysis

L02 cells were lysed using Protein Extraction Reagent (Invitrogen, Carlsbad, CA, USA) after treatment. The protein concentration was determined using the bicinchoninic acid (BCA) method. Equal amounts (40 μg) of total proteins were boiled for 5 min and subjected to sodium dodecyl sulfate-polyacrylamide gel electrophoresis (SDS-PAGE), followed by electrotransfer to polyvinylidene difluoride (PVDF) membranes. The membranes were blocked with nonfat dried milk for 3h at room temperature. Then the membranes were then incubated with β-actin (Abcam, Cambridge, UK), PPAR δ (Abcam, Cambridge, UK), insulin receptor substrate-1 [(IRS-1), Abcam, Cambridge, UK], phosphoinositide 3-kinase [(PI3K), Abcam, Cambridge, UK], phospho- protein kinase B [(AKT), CST, Beverly, MA, USA], p-AKT (CST, Beverly, MA, USA), Toll-like receptor 4 (TLR-4) [Proteintech, Rosemont, PA, USA], myeloid differentiation primary response gene 88 [(MyD88) Proteintech, Rosemont, PA, USA], inhibitor of nuclear factor kappa-B kinase α [(IKKα) CST, Beverly, MA, USA], p-IKKα (CST, Beverly, MA, USA), NF-κB1 (CST, Beverly, MA, USA), Bcl-2 (Proteintech, Rosemont, PA, USA), Bax (Proteintech, Rosemont, PA, USA), caspase-3 (Proteintech, Rosemont, PA, USA) and caspase-8 (Proteintech, Rosemont, PA, USA) antibodies (1:1,000 dilution) overnight at 4 ℃. The levels of β-actin were used as loading controls. The bands were visualized with an enhanced chemiluminescent direct labeling (ECL) system. The Gel Pro Analyzer 4.5 (Media Cybernetics; Silver Spring, MD, United States) was used to determine the band density. The quantification of band intensity was performed by using Quantity One software (Bio-Rad, CA, USA).

### Statistical Analysis

Statistical analyses of the results from the present study were performed using SPSS18.0. Data are expressed as mean ± standard deviation (SD). Statistical comparisons of the results were made by using analysis of variance (ANOVA). A level of *p* < 0.05 was considered statistically significant.

## Result

### The effect of PPAR δ on LPS-mediated lipid deposition progress of NAFLD

**Figure 1:**
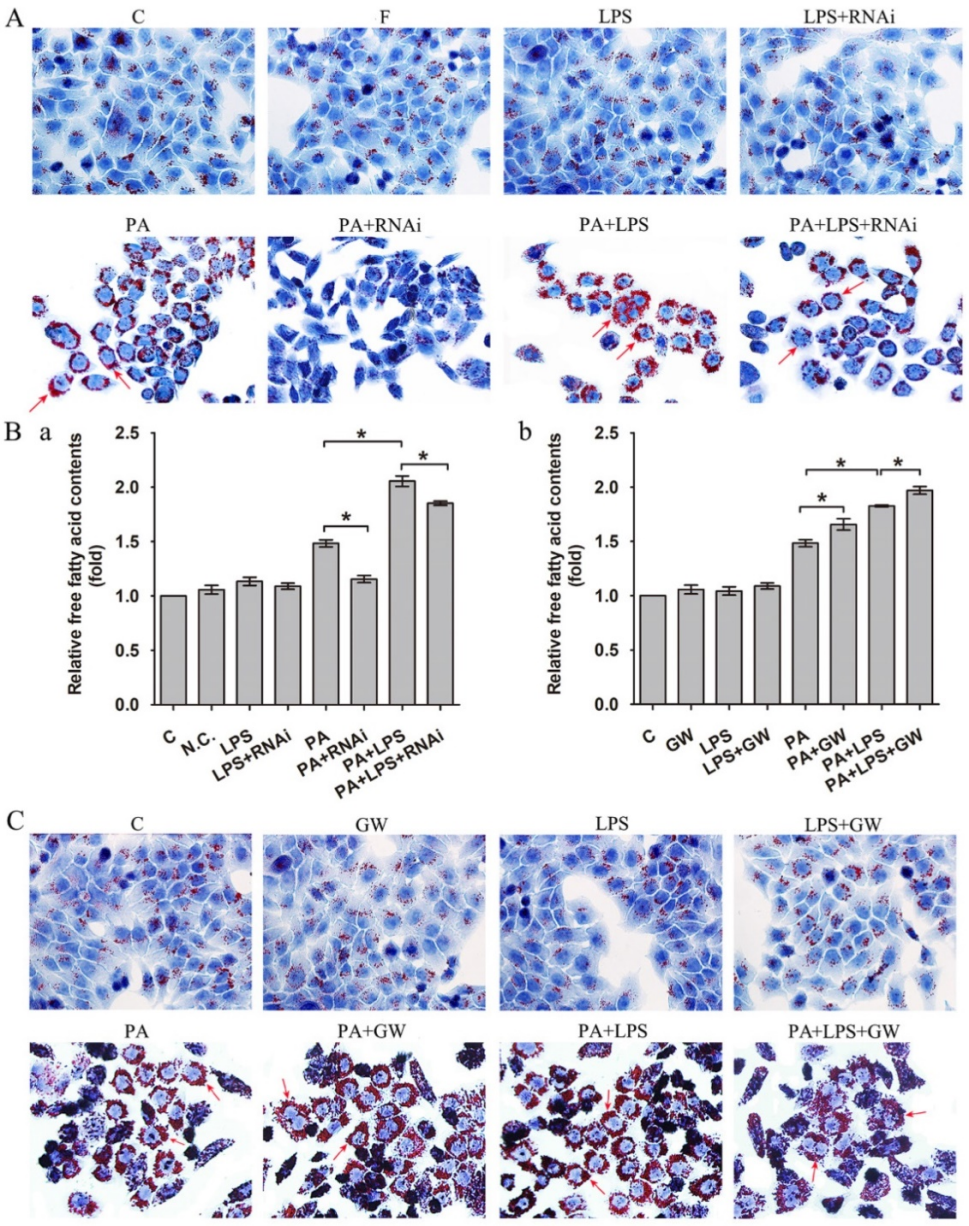
(A) Oil red O staining results of L02 cells were incubated in 0.4mM palmitic acid or (and) 800ng/ml LPS after N.C. siRNA or PPAR δ siRNA interference, arrowheads showed obvious red lipid droplets by Oil-red O stain (magnification, ×400). (B) The relative free fatty acid contents. (a) L02 cells were treated with N.C. siRNA or PPAR δ siRNA, (b) L02 cells were treated with or without GW0742 (*P<0.05). (C) Oil red O staining results of L02 cells were incubated in 0.4mM palmitic acid or (and) 800ng/ml LPS with or without GW0742 conditioning, arrowheads showed obvious red lipid droplets by Oil-red O stain (magnification, ×400). PA, palmitic acid. GW, GW0742.

As shown in Figure 1A, 800 ng/ml LPS treatments visibly raised intracellular lipid accumulation, with more lipid droplets than those of PA group. In addition, the FFA content measurement also suggested that 800 ng/ml LPS treatments upregulated the content of FFA (Figure 1Ba).

Then, we detected PPAR δ inhibition and activation on the impact of insulin resistance, since lipid accumulation and FFA expression are known to play critical roles in the insulin resistance. As shown in Figure 1A, si-PPAR δ treated group visibly had less lipid accumulation in cells. Meanwhile, the FFA content measurement also showed that si-PPAR δ treatments downregulated the content of FFA (Figure 1Ba). GW0742 sharply increased lipid accumulation (Figure 1C). In consistent with this, the content of FFA had a significant development in the GW0742 treated group (Figure 1Bb).

**Figure 2:**
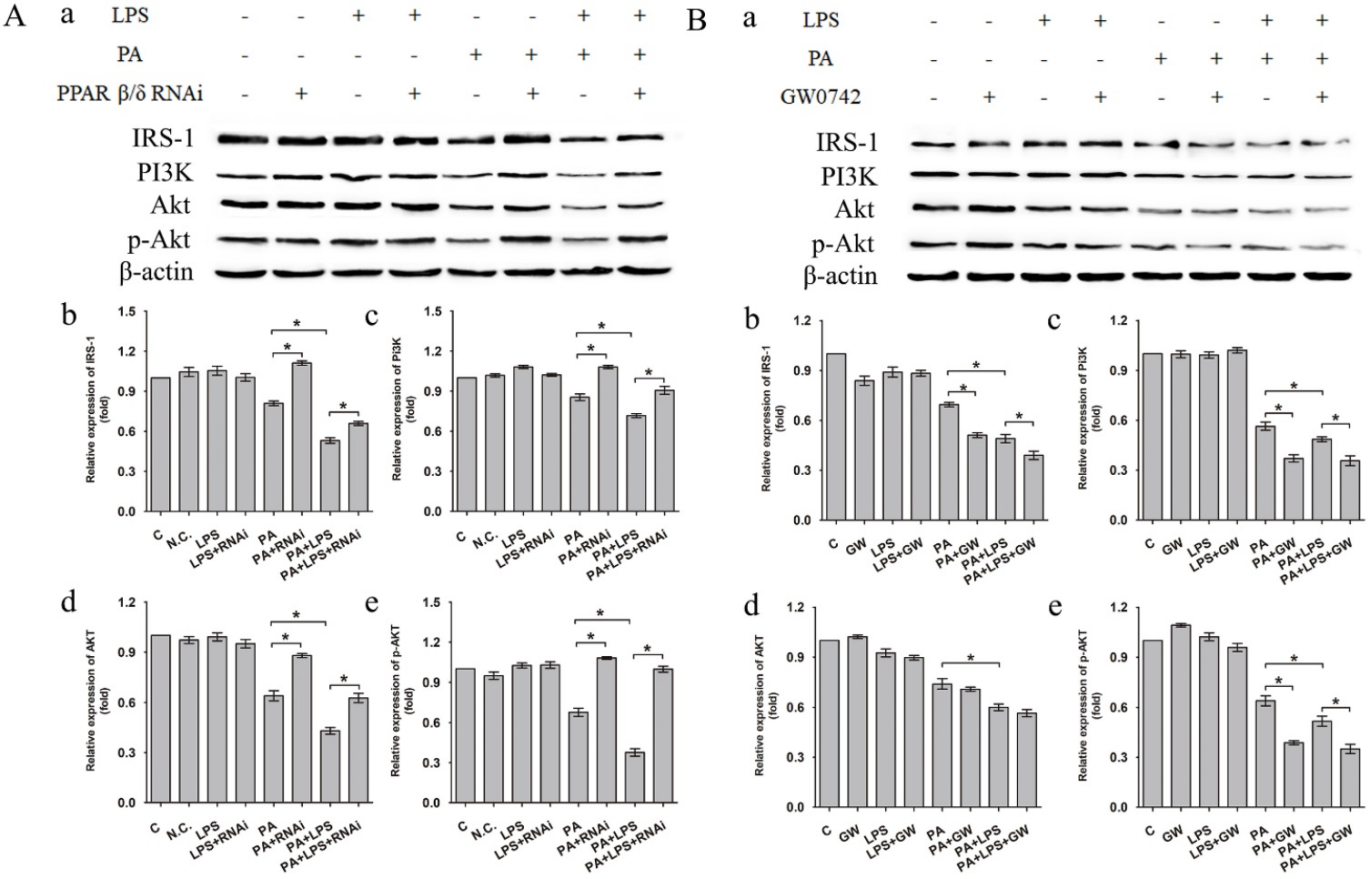
(**A**) Negative control siRNA (N.C. siRNA) or PPAR δ siRNA-transfected L02 cells were incubated in 0.4mM palmitic acid or (and) 800ng/ml LPS for 24h. (**a**) Relative expression level of IRS-1, PI3K, AKT and p-AKT were determined by Western blotting. (**b-e**) represent relative expression levels of IRS-1, PI3K, AKT and p-AKT. (**B**) L02 cells were exposed to 0.4mM palmitic acid or (and) 800ng/ml LPS with or without GW0742 treatment. (**a**) Relative expression level of IRS-1, PI3K, AKT and p-AKT were determined by Western blotting. (**b-e**) represent relative expression levels of IRS-1, PI3K, AKT and p-AKT. PA, palmitic acid. GW, GW0742.

The protein expression level of IRS-1, PI3K, AKT and p-AKT was significantly lower in PA+LPS group than in the PA group (Figure 2A a-e). Si-PPAR δ group showed a marked increase in the IRS-1, PI3K, AKT and p-AKT expression (Figure 2A a-e). On the contrary, GW0742 treated groups presented with lower protein expression levels of IRS-1, PI3K and p-AKT in comparison to the agonist untreated PA and PA+LPS group (Figure 2B a, b, c, e), whereas there was no change of AKT expression level between PA group and PA+GW group (Figure 2B d).

### The effect of PPAR δ on LPS-mediated expression levels of IL-6 and TNF-α

**Figure 3:**
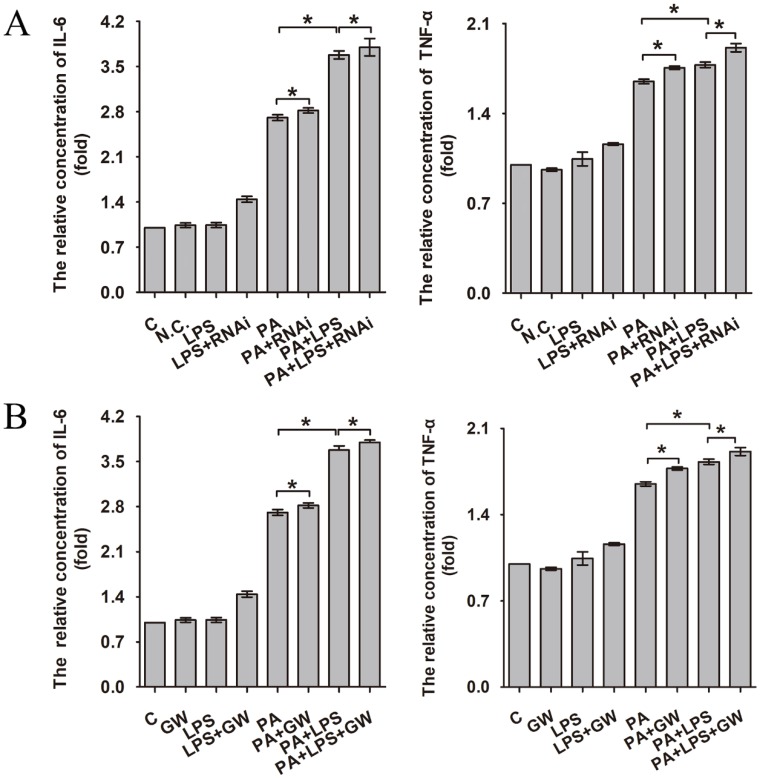
The relative concentration of IL-6 and TNF-α. (**A**) L02 cells were exposed to 0.4mM palmitic acid or (and) 800ng/ml LPS for 24h after N.C. siRNA or PPAR δ siRNA interference. (**B**) L02 cells were exposed to 0.4mM palmitic acid or (and) 800ng/ml LPS with or without GW0742 treatment. PA, palmitic acid. GW, GW0742.

Increased production of cytokines such as IL-6 and TNF-α is one of the earliest events in many types of liver injury 37. As can be seen in Figure 3A and B, 800 ng/ml LPS promotes the expression of IL-6 and TNF-α significantly, aggravating the level of inflammation. Compared with the PA group and LPS+PA group, si-PPAR δ downregulated the inflammatory cytokine level of IL-6 and TNF-α (Figure 3A). In contrast, GW0742 upregulated the level of IL-6 and TNF-α (Figure 3B), which suggests PPAR δ regulates inflammatory reaction.

**Figure 4:**
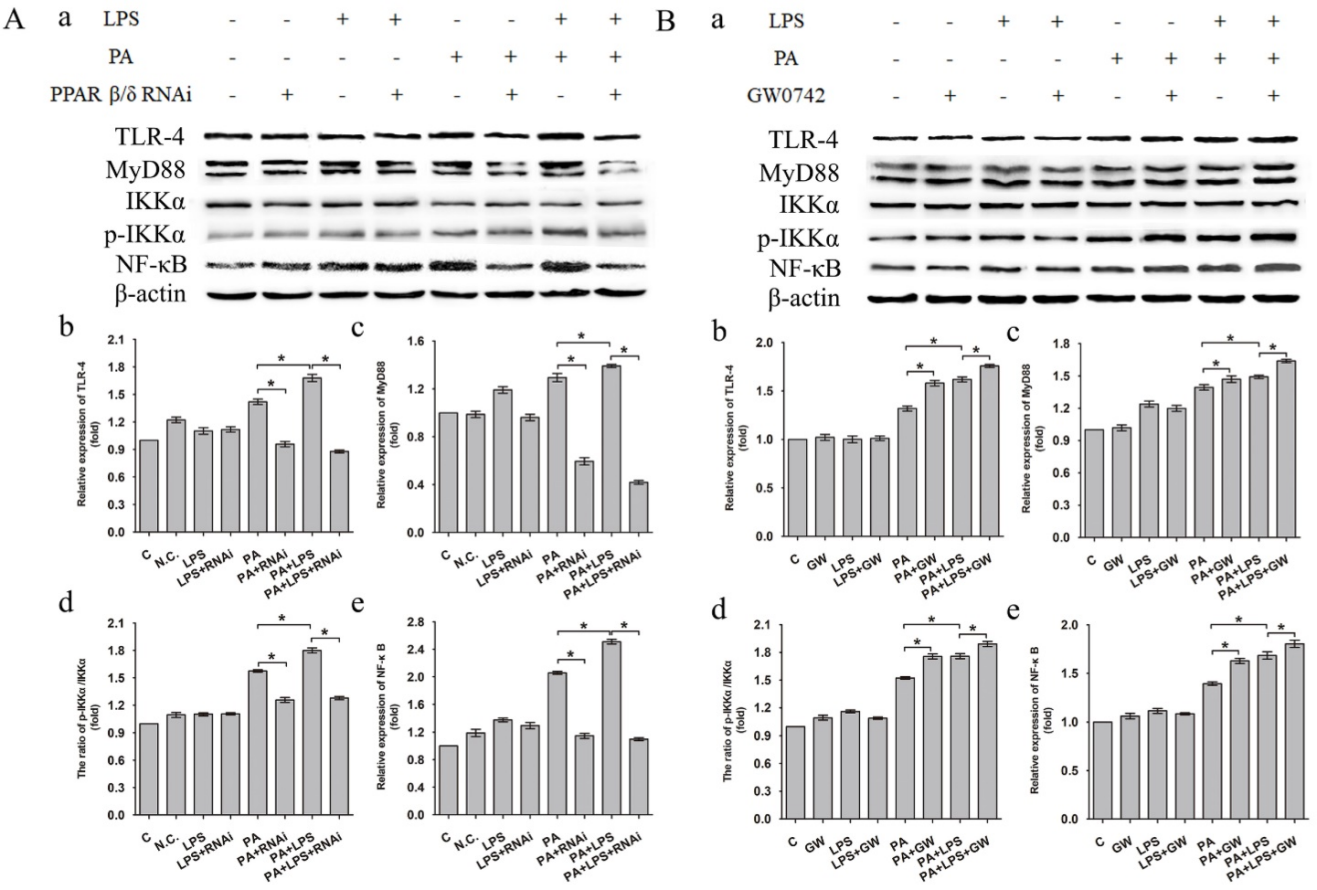
Expression level of TLR-4, MyD88, IKKα, p-IKKα and NF-κB after L02 cells incubated in 0.4mM palmitic acid or (and) 800ng/ml LPS for 24h with (**A**) L02 cells were N.C. siRNA or PPAR δ siRNA interference, (**B**) L02 cells were treated with or without GW0742 treatment (*P<0.05). PA, palmitic acid. GW, GW0742.

The expression level of TLR-4, MyD88 and NF-κB was increased in LPS+PA group than PA group (Figure 4A a, b, c, e). Treatment of si-PPAR δ caused a marked reduction in the expression levels of TLR-4, MyD88 and NF-κB in both PA and PA+LPS groups (Figure 4A b, c, e). In contrast, GW0742 caused increased expression levels of TLR-4, MyD88 and NF-κB in PA and PA+LPS groups (Figure 4B a, b, c, e). An increased phosphorylation level of IKKα was observed in LPS+PA group in comparison with PA group (Figure 4A d). As shown in Figure 4A d and Figure 4B d, treatment with si-PPAR δ reduced the phosphorylation level of IKKα in both PA and PA+LPS groups, whereas the treatment with GW0742 caused a markedly increased expression level of IKKα in both PA and PA+LPS groups.

### The effect of PPAR δ on LPS-mediated apoptosis in NAFLD

**Figure 5:**
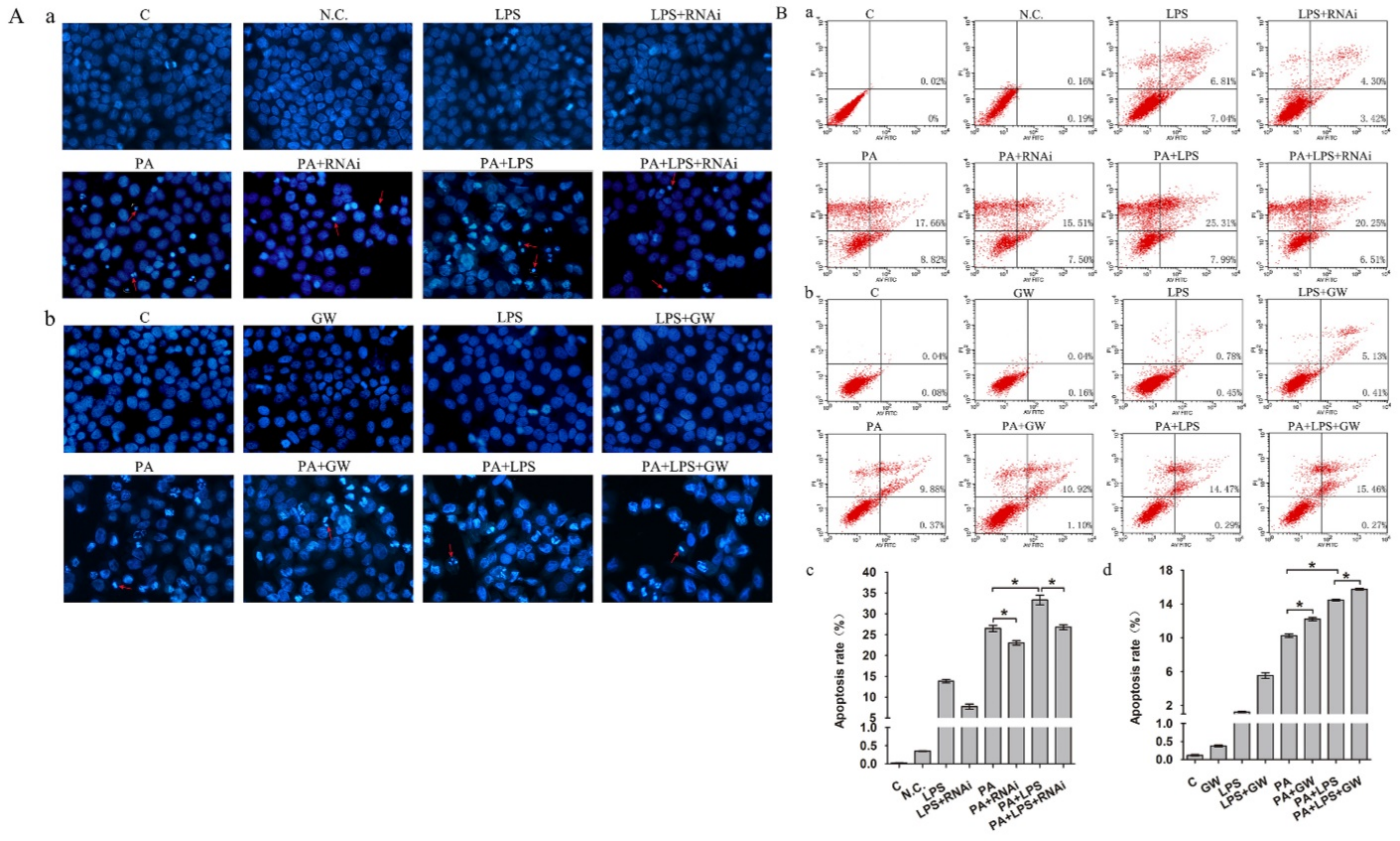
(**A**) Hoechst 33342 staining results of L02 cells. (**a**) L02 cells were exposed to 0.4mM palmitic acid or (and) 800ng/ml LPS for 24h after N.C. siRNA or PPAR δ siRNA interference (magnification, ×400). (**b**) L02 cells were exposed to 0.4mM palmitic acid or (and) 800ng/ml LPS for 24h with or without GW0742 treatment (magnification, ×400). (**B**) Detection of apoptotic cells by Annexin V/PI double-staining. (**a**) L02 cells were treated with N.C. siRNA or PPAR δ siRNA interference and (**b**) L02 cells were treated with or without GW0742 treatment. Quantitative analysis of apoptosis. (**c**) N.C. siRNA or PPAR δ siRNA interference. (**d**) L02 cells were treated with or without GW0742 treatment (*P<0.05). PA, palmitic acid. GW, GW0742.

Gut microbiota could lead to an excess accumulation of lipid in the hepatocytes, which result in lipotoxicity and trigger hepatocyte cell death in NAFLD 38. To elucidate the effects of LPS on apoptosis in FFA treatment L02 cells, Hoechst 33342 staining and Annexin V-FITC/PI double staining were performed. Results of Hoechst 33342 staining showed that the chromatin condensation and number of apoptotic bodies were increased in PA+LPS treated group compared to those of PA group (Figure 5A). Similarly, LPS treatment significantly augmented the percentages of apoptotic cells compared with the PA group (Figure 5B). Hoechst 33342 staining demonstrated that the chromatin condensation and number of apoptotic bodies were decreased in si-PPAR δ treated groups (Figure 5Aa). PPAR δ inhibition group' percentage of early and late apoptotic cells is significantly lower than those of the PA group and LPS+PA groups, suggesting that apoptosis of NAFLD was reduced by PPAR δ inhibition (Figure 5B a, c). By contrast, GW0742 treatment significantly augmented the number of apoptotic bodies and percentages of apoptotic cells compared with the PA and PA+LPS groups (Figure 5Ab and Figure 5B b, d).

**Figure 6:**
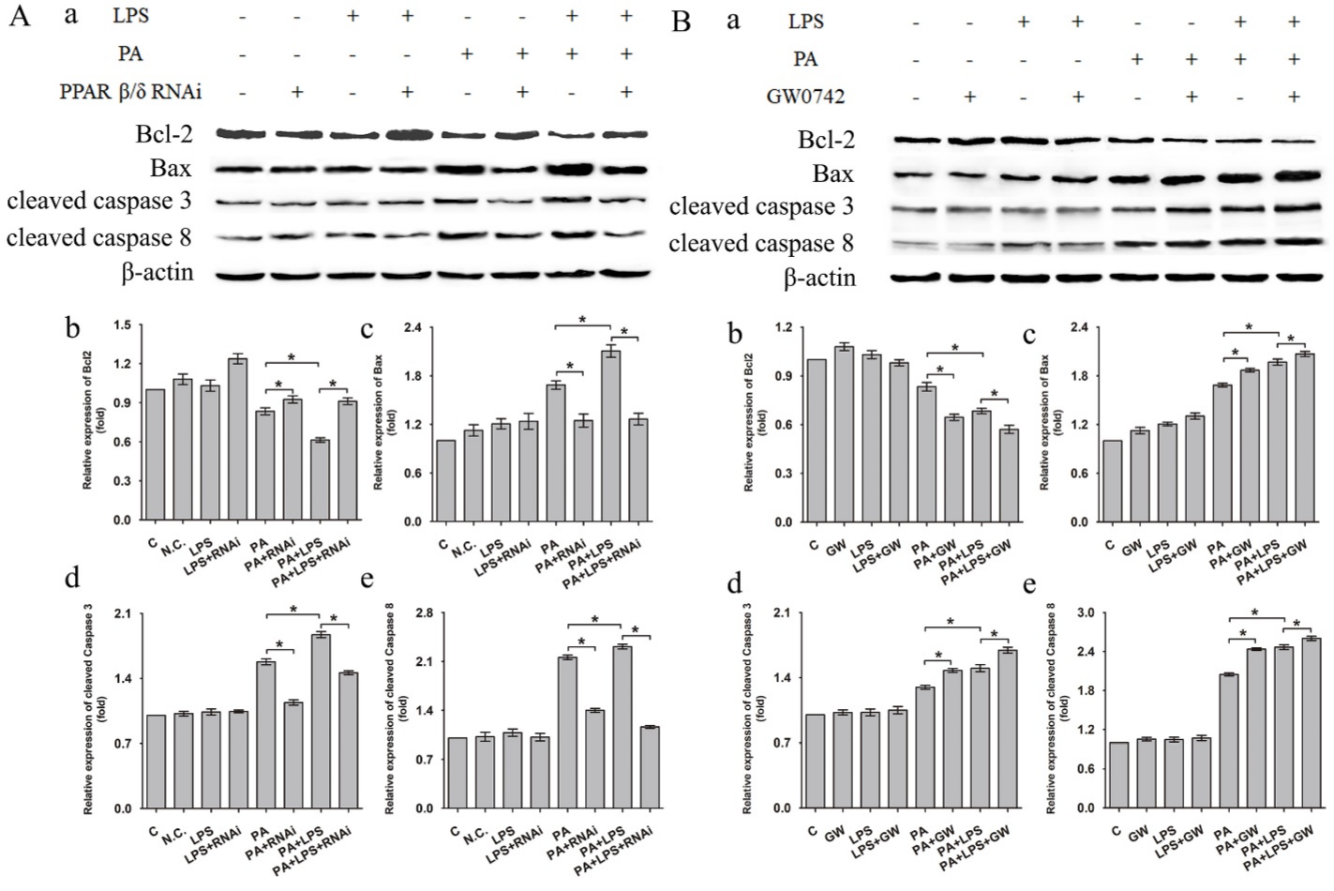
Expression level of Bcl-2, Bax, cleaved Caspase 3, and cleaved Caspase 8 after were exposed to 0.4mM palmitic acid with or without 800ng/ml LPS for 24h (**A**) L02 cells were treated with N.C. siRNA or PPAR δ siRNA interference or (**B**) L02 cells were treated with or without GW0742 treatment. PA, palmitic acid. GW, GW0742. PA, palmitic acid. GW, GW0742.

Western blotting analysis showed that related proteins during caspase signal transduction were detected. As shown in Figure 6, ​the expression levels of Bax, cleaved caspase 3, and cleaved caspase 8 were elevated after LPS treatment for in PA group. si-PPAR δ treatment showed obvious reduction in expression levels of Bax, cleaved caspase 3, and cleaved caspase 8 (Figure 6A a, c, d, e). Conversely, the GW0742 treated groups upregulated the ​expression levels of Bax, cleaved caspase 3 and cleaved caspase 8 expression when compared to PA and PA+LPS control groups (Figure 6B a, c, d, e). The expression trend of Bcl-2 was contrary to the above results of Bax, cleaved caspase 3, and cleaved caspase 8 after treatment by LPS, si-PPAR δ, and GW0742 (Figure 6Ab and Figure 6Bb). The result of si-PPAR δ and GW0742 treatment were completely consistent to the results of cleaved caspase 3 and cleaved caspase 8 expression, suggesting PPAR δ inhibition reduced apoptosis caused by FFA and LPS in L02 cells.

## Discussion

The mechanism for the development and progression of NAFLD is complex and multifactorial 36, 39-43. The 'two hits' hypothesis cannot illuminate the pathological mechanism of NAFLD completely. By literature review, we assume that LPS is a pathogenic factor of NAFLD. Nevertheless, there is few studies to assess the role of PPAR δ in LPS-associated NAFLD. The present study was designed to explore the possible effects of LPS on NAFLD, and to explore the effect of PPAR δ in this process.

NAFLD is the accumulation of fat in the hepatocytes, in the form of lipid droplets containing triglycerides (TG). Increased delivery of FFA, intrahepatic de novo lipogenesis, and dietary fat are the major mechanisms underlying TG accumulation 44. In the present study, oil red staining indicated that LPS enhanced the accumulation of TG in L02 cells, comparing with PA treatment (NAFLD model). And the FFA assay results were consistent with those of oil red staining. PPAR δ inhibition visibly reduced the LPS-induced TG accumulation and downregulated the content of FFA compared with PA treated and PA+LPS treated groups, whereas PPAR δ activation showed the reverse trend. These results suggest that LPS promotes lipid deposition in hepatocytes and PPAR δ inhibition may be a protective strategy against LPS-associated lipid accumulation.

Increased intracellular lipid accumulation and levels of serum FFA are due to a failure of insulin-mediated suppression of lipolysis, and being important mediators of insulin resistance 45-47. Several studies showed that elevated serum levels of LPS may induce hepatocyte steatosis by increasing insulin resistance 27-30. IRS-1 is the major insulin receptor effector that responsible for the transduction of insulin signaling 48. And, multiple evidences have suggested that deregulation of the PI3K/AKT signaling pathway is a vital molecular mechanism underlying the insulin resistance 49, 50. Data obtained from western blotting showed that LPS decreased the relative expression levels of IRS-1, PI3K, AKT and phosphorylation of AKT, contributing to insulin resistance. Simultaneously, PPAR δ inhibition improved LPS-induced depression of the expression of IRS-1, PI3K, AKT and phosphorylation of AKT. Instead, PPAR δ agonist showed the reverse pattern. The evidence above indicated that LPS may affect insulin resistance through IRS-1/PI3K/AKT pathway, thereby regulating the hepatic lipid accumulation. Meanwhile, PPAR δ inhibition improves LPS-induced lipid deposition and insulin resistance through regulating IRS-1/PI3K/AKT pathway. Furthermore, PPAR δ might improve insulin resistance via reducing the delivery of free fatty acids to the liver, promote FFA β-oxidation, and diminish de novo lipogenesis 51.

Intestinal dysbiosis causes increased intestinal permeability and gut-liver axis dysfunction, leading to increased risk of endotoxemia, followed by the activation of proinflammatory pathways after binding with specific hepatic receptors 52. Hence, downregulating inflammation level of liver may relieve insulin resistance in NAFLD 53, 54. Our results showed that LPS upregulated the expression level of two important pro-inflammatory cytokines, IL-6 and TNF-α, through interacting with its principal ligand TLR-4, initiating a TLR4/MyD88/NF-κB signalling cascade culminating in a pro-inflammatory response. Our results are consistent with previous findings 55-59. In addition, FFA could also activate the pro-inflammatory pathways through membrane receptors. Our result revealed that PPAR δ inhibition reduced the LPS-induced cytokine expression, such as IL-6 and TNF-α, and secretion by preventing activation of phosphorylation of IKKα and NF-κB, which reduces the inflammation. Moreover, PPAR δ inhibition probably induces FFA β-oxidation, reducing their availability for the synthesis of deleterious complex lipids involved in inflammation. Consistent with previous studies, our findings reveal that PPAR δ could regulate expression of inflammation factors 60, 61.

**Figure 7:**
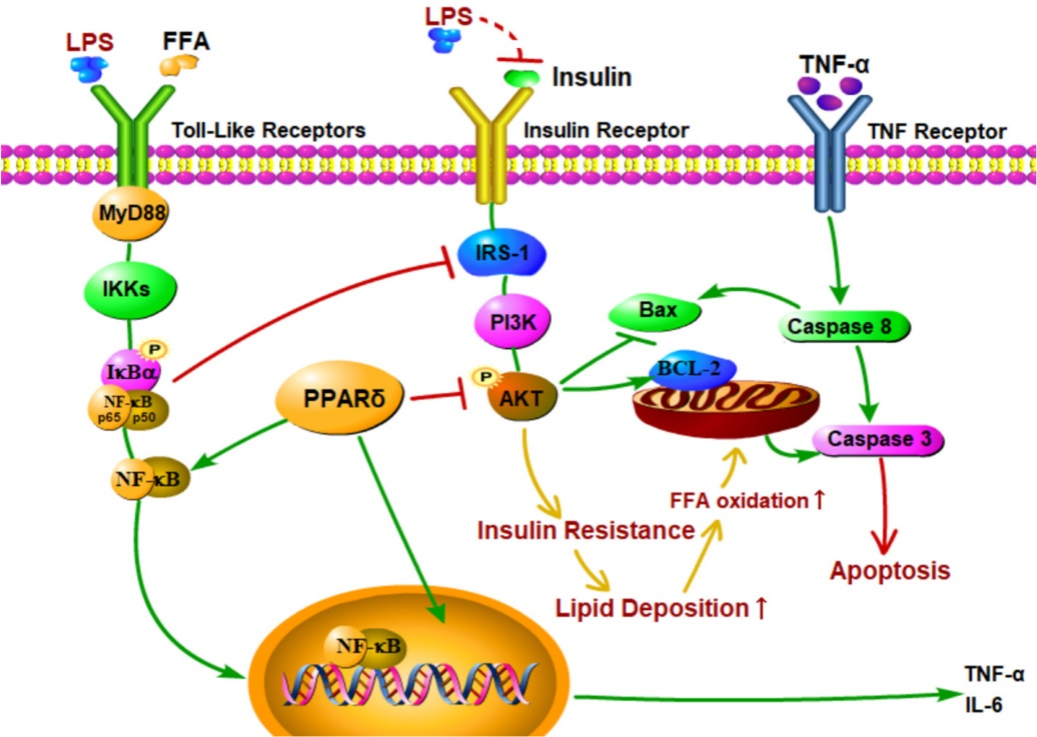
Proposed mechanisms for PPAR δ inhibition protects against palmitic acid-LPS induced lipidosis and injury in L02 cell by adjusting lipid accumulation, insulin resistance, pro-inflammation and apoptosis-associated proteins.

Recent studies showed that the increased levels of LPS and proinflammatory cytokines led to liver histological changes, such as hepatocyte necrosis and apoptosis 62-64. In this study, both Annexin V-FITC/PI double staining and Hoechst 33342 staining showed that LPS induced apoptosis in the PA treated group. In addition, LPS and PA treatment upregulated expression levels of apoptotic proteins, such as Bax, cleaved caspase 3, and cleaved caspase 8, and downregulated the expression level of Bcl-2 compared with the PA groups. These findings suggest that LPS induce cell apoptosis in NAFLD L02 cell through a mitochondrial apoptotic pathway and caspase-dependent pathway. These results may be due to the enhanced level of FFA and TNF-α 65-67. Excessive increase of FFAs may result in an increase of mitochondrial damage, resulting in cascade reaction of caspase to induce cell apoptosis 68, 69. Furthermore, TNF-α might activate caspase 8 relevant apoptotic pathway through the activation of death receptor (Figure 7). Meanwhile, PPAR δ inhibition alleviated LPS-induced cell apoptosis with depressed protein expression level of cleaved caspase 3, cleaved caspase 8 and Bax in L02 cells. On the contrary, PPAR δ activation showed the reverse trend. The results indicate that PPAR δ plays an important role in LPS-induced apoptosis of NAFLD L02 cell. PPAR δ inhibition probably prevents mitochondria releasing apoptotic factors by upregulating the expression of PI3K/AKT and reduces lipidtoxicity caused by increased FFA.

PPAR δ play a crucial role in LPS mediated insulin resistance, inflammation and apoptosis in NAFLD L02 cell line. Several studies showed that the PPAR δ activation modulated multiple parallel paths to decrease the hepatic inflammation, lipotoxicity, insulin resistance and, therefore, mitigate the LPS induced progress of lipid deposition in HepG2 cells and mouse primary liver cells 70, 71. In contrast, other data indicate that the PPAR δ activation did not act as protective effects as described. In line with our results, adenovirus-mediated hepatic PPAR δ over-expression has been shown to activate de novo lipogenesis and subsequent lipid deposition 72. GW501516 treated* db/db* mice exhibited higher expression of the lipogenic enzyme acetyl-CoA carboxylase-β and elevated TG levels in the liver 73. In PPAR -/- mice, there are no changes in cholesterol, TG, high density lipoprotein cholesterol and FFA with a normal chow diet and there is no data for lipoprotein profiles during feeding by high-fat diets 74. Therefore, the role of PPAR δ is still controversial in the repair process of NAFLD. There are several possible reasons explainning the observed differences. One crucial factor is due to dissimilarities in PPAR δ tissue expression patterns among human, mouse and rat 75-77. Besides, PPAR δ is widely expressed in many organs 31, 78, So, PPAR δ activation might increase fatty acid oxidative capacity and energy dissipation in skeletal muscle cells, thus improving hepatic steatosis. Last but not the least, short-term treatment with PPAR δ agonists reportedly yields a transient increase in hepatic TG levels 78.

To summarize, LPS may be a key factor in development of NAFLD, and PPAR δ inhibition protects the liver in case of the NAFLD progression, which provides a new evidence of PPAR δ in the molecular pathogenesis of LPS-related NAFLD damage. Due to limited time and energy, in vivo experiment is not executed. We are conducting experiments in vivo to compensate for the insufficient evidence, *in vitro*. All authors read and approved the final manuscript.

## Abbreviations

NAFLD: Non-alcoholic fatty liver disease
NASH: non-alcoholic steatohepatitis
HCC: hepatocellular carcinoma
IR: insulin resistance
LPS: lipopolysaccharides
TLR-4: Toll-like receptor 4
PPAR δ: Peroxisome proliferator-activated receptor δ
FFA: free fatty acid
HDL: high-density lipoprotein
