# PPAR δ inhibition protects against palmitic acid-LPS induced lipidosis and injury in cultured hepatocyte L02 cell: Erratum

**DOI:** 10.7150/ijms.91745

**Published:** 2023-11-29

**Authors:** Yi Li, Chenwei Wang, Jiyuan Lu, Ke Huang, Yu Han, Junlin Chen, Yan Yang, Bin Liu

**Affiliations:** 1 School/Hospital of Stomatology, Lanzhou University, Lanzhou, China; 2 College of Life Science & Technology, Huazhong University of Science and Technology, Wuhan, China; 3 Department of Endocrinology, Gansu Provincial Hospital, Lanzhou, China

When reviewing the previous work, we recognized the images of the original Figure 1 of our article were incorrectly assembled. The image of the LPS group in the original Figure 1A was misused due to a mislabeling error. We express our sincerest apologies to readers and researchers who have any inconvenience caused by this error.

Figure 1 should be corrected as follows. The authors confirm that the correction made in this erratum does not affect the original conclusions.

## Figures and Tables

**Figure 1:**
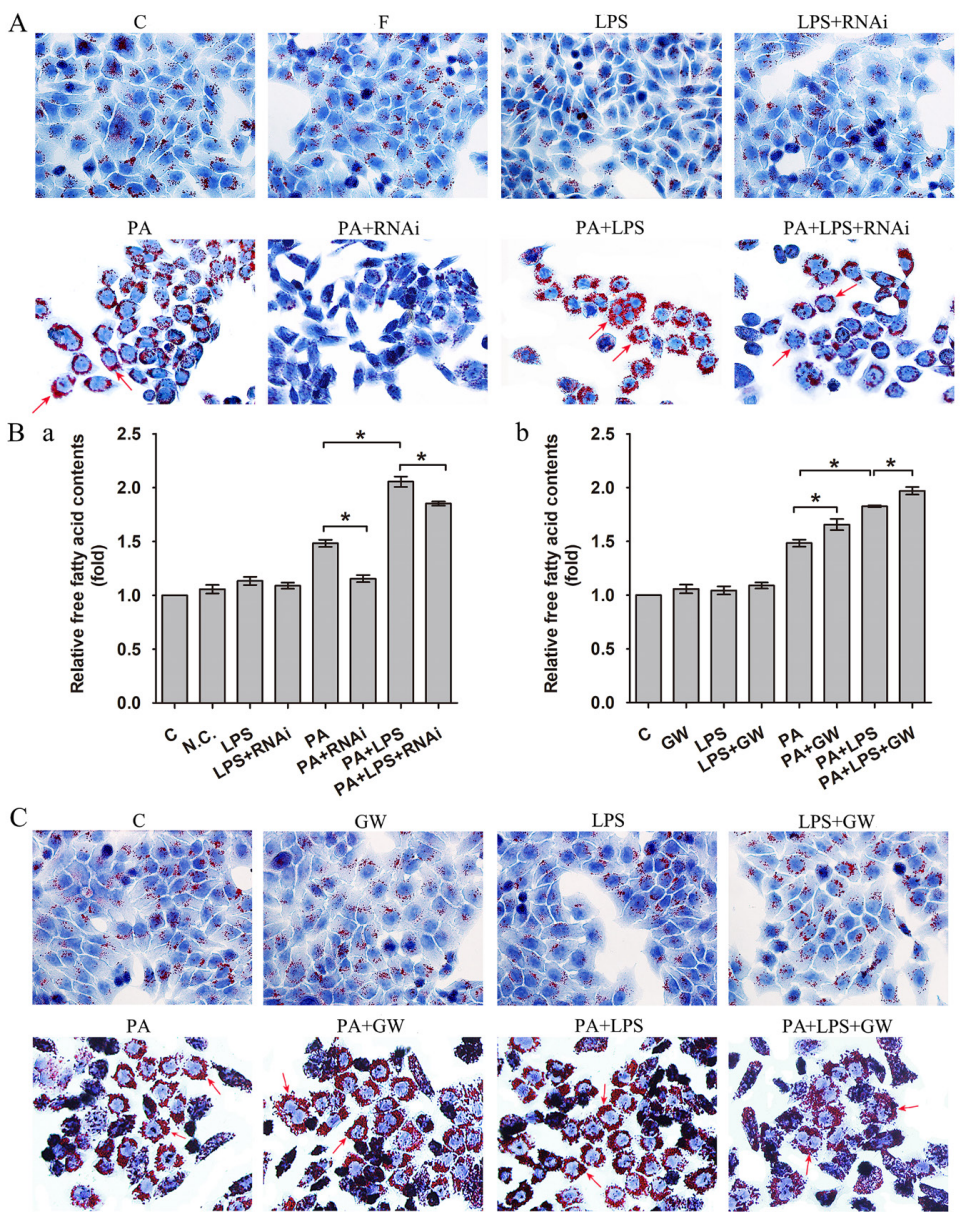
(A) Oil red O staining results of L02 cells were incubated in 0.4mM palmitic acid or (and) 800ng/ml LPS after N.C. siRNA or PPAR δ siRNA interference, arrowheads showed obvious red lipid droplets by Oil-red O stain (magnification, ×400). (B) The relative free fatty acid contents. (a) L02 cells were treated with N.C. siRNA or PPAR δ siRNA, (b) L02 cells were treated with or without GW0742 (*P<0.05). (C) Oil red O staining results of L02 cells were incubated in 0.4mM palmitic acid or (and) 800ng/ml LPS with or without GW0742 conditioning, arrowheads showed obvious red lipid droplets by Oil-red O stain (magnification, ×400). PA, palmitic acid. GW, GW0742.

